# Development and validity of a hazard prediction test for Chinese drivers

**DOI:** 10.1371/journal.pone.0245843

**Published:** 2021-01-25

**Authors:** Bocong Wu, Long Sun, Na Gu

**Affiliations:** School of Psychology, Liaoning Normal University, Dalian, Liaoning, P. R. China; Monash University, AUSTRALIA

## Abstract

**Background:**

Hazard perception ability, which develops with driving experience, has been proven to be associated with drivers' traffic involvement. Although classic reaction time-based hazard perception tests have been developed in many developed counties, experience-related differences may not be found in drivers from developing countries due to their increased opportunities to experience hazards on roads. Therefore, the present study aims to develop a hazard prediction test for Chinese drivers based on a predictive paradigm called “What happens next?” and assess its reliability and validity.

**Method:**

Thirty-six video clips filmed from drivers’ perspectives of Chinese driving settings were presented to 54 novice drivers and 47 experienced drivers. Participants were asked to answer three questions after each video clip was blacked out and to then quickly press the mouse button on a reaction time-based hazard perception test. Both the differences in the test scores between novice and experienced drivers and the differences in scores between drivers with and without traffic violations were compared.

**Results:**

The final hazard prediction test consisted of 20 video clips. A high internal consistency coefficient of the test, i.e., Cronbach's alpha = 0.862, was obtained. The total scores of the test were positively and significantly correlated with reaction times as measured on the video-based hazard perception test, thus providing evidence regarding the discriminant validity of the test. More importantly, drivers with traffic violations obtained significantly lower total scores on the test than did drivers without traffic violations.

**Conclusion:**

The newly developed hazard prediction test exhibited adequate psychometric properties and provided a practical alternative for assessing drivers’ hazard perception ability in China.

## 1. Introduction

The number of traffic accidents in developing countries accounts for 80% of the world’s road traffic accidents [1]. According to the data from the National Bureau of Statistics of China [2], a total of 244,937 cases resulted in 63,000 deaths and caused immeasurable losses in 2018. Since human factors account for approximately 90% of accidents, many countries have rendered hazard perception tests a compulsory part of driving tests. Hazard perception refers to the ability of drivers to anticipate potential road hazards [3]. Numerous studies have found that young novice drivers' response times to hazards in video clips are slower than those of experienced drivers [4,5], albeit with some exceptions [6,7]. More importantly, the slower response times of young novice drivers are associated with their traffic crashes [8,9]. Thus, reaction time-based hazard perception tests have been developed for driver testing and training in some developed countries, such as Australia [4] and England [10]. However, hazard perception tests have not been widely used in driver testing or training in developing countries, such as China.

Traditional hazard perception tests are typically based on the reaction time paradigm using still images [11], simulated driving situations [12,13], road tests [9] and clips of normal driving [3]. In these tests, drivers are instructed to press a button or touch the screen once they detect a potential developing hazard. Although many studies have found that hazard perception ability develops over time with driving experience, there are studies that have revealed no experience-related difference between driver groups [6,7]. For instance, similar response latencies in video clips were found between novice drivers and experienced drivers [7]. One possible explanation for this discrepancy is the methodology used in these studies [12,14].

Cross-cultural studies on drivers in England and Malaysia indicate that the reaction time-based hazard perception test is less effective in discriminating between novice and experienced drivers in developing countries [15]. The underlying reason for this may be that drivers in developing countries have more opportunities to experience road hazards. Thus, a road hazard that was identified by a driver from a developed country may not be hazardous enough for a driver from a developing country, such as China. That is, hazard desensitization may increase drivers’ risk thresholds, thus making drivers with varying experiences reluctant to respond, i.e., press the button [14,16,17]. In this case, the reaction time obtained from the test may have little to do with the hazard perception ability but rather be more related to the driver’s risk acceptance [12].

Although the reliability of the traditional hazard perception test is acceptable and the test can distinguish between novice and more experienced drivers, many researchers doubt its validity, claiming that it may not test the driver’s ability to identify hazards and anticipate how they (the hazards) will develop [14,18]. Accordingly, lower test scores on reaction time-based tests may be arbitrary as a lower score could either reflect a proficient ability to perceive what constitutes a hazard or indicate a poor performance on the test [10]. To provide an alternative to assess drivers’ hazard perception ability, several researchers attempted to develop a hazard prediction test using a predictive paradigm [1,10,19] that originated from situation awareness (SA) theory. Correspondingly, several researchers have defined hazard perception as situation awareness on the road or the ability to read the road [20]. Within the SA model, there are three levels, namely, perception (level 1), comprehension (level 2), and projection (level 3) [21], which respectively approximate the source, location, and projection of the road hazard. A number of studies have applied the SA model to hazard prediction assessments and found that it successfully discriminates among driver groups with varying experience [19,22–24].

The predictive paradigm, also called the “what happens next?” (WHN) method, uses three questions to assess drivers’ hazard perception ability. The three questions are “What is the hazard?” (question 1), “Where is the hazard?” (question 2) and “What happens next?” (question 3). During the experiment, participants are required to select a response from the given choices, or orally respond to questions when a video clip is cut to black or the final still image of the clip remains on the screen [10,18,22]. Compared to the reaction time paradigm, the WHN method can prevent the adverse effects of risk threshold and response criterion on participants’ response [16,25]. On the other hand, as the hazard in the video clip is normally at the very beginning, it does not develop into an acute hazard, which causes the participants’ responses to be more reliant on their visual detection and anticipation of the hazards [12].

One study developed a hazard prediction test based on the WHN method found that experienced drivers anticipated more hazards correctly than did novice drivers when the screen went black [10]. Another study compared the hazard perception ability of drivers from Malaysia and the UK using a predictive paradigm and found that the clips filmed in Malaysia could not differentiate among participants with respect to experience and that Malaysian drivers are less accurate in predicting hazards than are British drivers [1]. However, the study did find that experienced Malaysian drivers outperformed novice drivers when comparing test scores. Similarly, a third study found that Spanish learner drivers obtained the lowest scores on a hazard prediction test compared to novice and experienced drivers [19]. Hence, one can conclude that these studies provide robust evidence for the validity of the hazard prediction test. Thus, given that road casualties are a major problem in China, the present study develops a hazard prediction test and compares its validity with a reaction time-based hazard perception test.

Another issue that must be addressed in the literature is whether the scores on a hazard prediction test predict drivers’ traffic violations. Regarding reaction time-based hazard perception tests, only a few studies have found a positive, albeit but weak, association between drivers’ slower reaction times and certain types of traffic crashes [8]. There are studies that have also examined the associations between hazard perception ability and crash risk by measuring drivers’ situation awareness [26]. However, to our knowledge, only two studies have examined the relationship between the scores obtained on a WHN-based hazard perception test and the number of traffic violations. These studies found that drivers with multiple offenses detected hazards well, but they underestimated the risks involved and were less cautious than non-recidivist drivers [19,22]. Given that traffic crashes are rare, this study will examine the relationship between the scores on the hazard prediction test and the number of traffic violations the participants received in the past 12 months to further examine the validity of the prediction test.

The main purpose of the present study is to develop a hazard prediction test using the WHN method and assess its reliability and validity among Chinese drivers. A secondary purpose of this study is to examine whether the test scores are associated with traffic violations. The results of the study can be used to develop interventions and training for Chinese drivers.

## 2. Methods

### 2.1. Participants

This study was approved by the Logistics Department of the Civilian Ethics Committee of Liaoning Normal University and was conducted from July 1 to 15, 2019. The participants included 104 drivers, all of whom signed an informed consent form. The sample was composed of private car drivers from Dalian (northeast of China), Shanghai (southeast of China), Zhengzhou (center part of China) and Kunming (southwest of China), which are four cities that geographically well represent China. Three experienced drivers withdrew from the experiment at the halfway mark. Thus, a total of 101 drivers finished the experiment with intact data. The sample was composed of 57 females and 44 males, ranging in age from 19 to 45 years old (*M* = 26.41, *SD* = 6.25). Participants’ years of driving experience ranged from six months to 12 years (*M* = 2.72, *SD* = 2.35). With respect to education, 10.9% of the participants held primary school certificates, 37.6% held secondary certificates, and 51.5% held university certificates.

In the present study, 54 drivers belonged to novice driver groups (32 females) and 47 drivers belonged to experienced driver groups (25 females), based on their previous driving experience (see Table 1). Novice drivers were drivers who had less than two years of driving experience, and experienced drivers had a minimum of three years of driving experience. Of the participants, 23 drivers reported having at least one traffic violation while driving during the past year, whereas the other 78 drivers reported no traffic violations while driving during the past year. The demographic information of the drivers with and without traffic violations is presented in Table 2.

**Table 1:** Demographic information of novice and experienced drivers (*M±SD*).

Demographic information	Novice drivers (*n* = 54)				Experienced drivers (*n* = 47)			
	Min	Max	*Mean*	*SD*	Min	Max	*Mean*	*SD*
Age (yr)	19	27	21.93	2.20	25	45	31.55	5.35
Years of driving experience (yr)	0.5	2	1.10	0.58	3	12	4.64	2.18
Kilometers driven per year (km)	15	6000	666.94	1210.14	200	50000	6653.40	12429.10
Years of education (yr)	9	16	14.81	2.56	9	16	12.49	1.77
Driving frequency ^a^	1	4	2.83	0.79	1	3	1.87	0.74
Number of violations last 12 months	0	2	0.24	0.51	0	2	0.32	0.59
Number of crashes last 12 months	0	1	0.06	0.23	0	1	0.06	0.25

^a^ 1 = daily, 2 = weekly, 3 = monthly, 4 = rarely.

**Table 2:** Demographic information of drivers with and without traffic violations (*M±SD*).

Demographic information	Drivers with violations(*n* = 23)				Drivers without violations(*n* = 78)			
	Min	Max	*Mean*	*SD*	Min	Max	*Mean*	*SD*
Age (yr)	19	30	24.91	3.38	19	45	26.85	6.82
Years of driving experience (yr)	1	6	2.78	1.40	0.5	12	2.74	2.56
Kilometers driven per year (km)	50	10000	2069.57	2848.31	15	50000	3860.58	10096.23
Years of education (yr)	9	16	12.91	2.78	9	16	13.97	2.38
Driving frequency ^a^	1	4	2.22	0.95	1	4	2.44	0.89
Number of violations last 12 months	1	2	1.22	0.42	0	0	0	0
Number of crashes last 12 months	0	1	0.13	0.34	0	1	0.04	0.19

^a^ 1 = daily, 2 = weekly, 3 = monthly, 4 = rarely.

### 2.2. Materials

#### 2.2.1 Hazard perception video

Three hundred video clips were recorded in urban and rural areas of Dalian between June and September of 2018 using an HP F300 Car Camcorder, and all clips were shot from the drivers' perspective under either sunny or cloudy conditions. The original video was clipped using the Solveig MM Video Splitter 4.0. Two experts in traffic psychology were asked to evaluate each clip, and clips that were determined to be overly complicated or to have incomprehensible backgrounds, vague sources of danger, or more than one source of danger were deleted as were clips that could easily cause ambiguity or confusion for the driver. The result following the evaluation of the experts was an initial test comprised of 42 clips.

The stopping point for each hazard was then identified by another two experts. As in the previous study [10], the stopping point occurred immediately before the actual hazard unfolded (see Appendix A for a description of each clip). Seven clips were discarded due to an interrater reliability score below 0.70. The remaining 35 clips were used for the experiment. Clip length varied from 9 s to 16 s (*M* = 12.54, *SD* = 2.54), with each clip presenting a driving scenario that contained a slowly developing hazard. A hazard was defined as any obstacle that required drivers to perform an evasive maneuver to avoid a collision [27]. However, drivers did not need to actually respond because the developing hazards did not develop into acute hazards [10,19].

#### 2.2.2 Hazard prediction test

As in previous studies [10,19,22], the present study occludes mere hundreds of milliseconds after hazard onset, and black screens appear immediately upon the occlusion of the clips. After the clips are occluded, participants are asked to answer the following questions: “What is the hazard?” (question 1), “Where is the hazard?” (question 2), “What happens next?” (question 3). For each question, participants received two points for a correct answer, one point for a partially correct answer, and 0 points for a wrong answer. The final score is the sum of the three questions. When the video clips stopped, the participants were asked to write their answers to each of the three questions.

The final answers to the questions were confirmed and evaluated by two expert drivers, and Cronbach's α was calculated to assess the interrater reliability [19]. A high degree of consensus was achieved on each of the questions (question one κ = 0.81, question two κ = 0.86, question three κ = 0.82; total score κ = 0.80).

#### 2.2.3 Video-based hazard perception test

The test contained 12 clips [28]. All of the clips were filmed in April and May of 2016 under either sunny or cloudy weather conditions. Each clip included only one slowly developing hazard under varying circumstances situations (see Appendix B for a description of each clip). The appearance time and location of the hazard in each clip were random from one clip to another the hazard triggers in the video clips included cars, motorcycles, cyclists, road work and pedestrians, with the clip length ranging from 10 s to 16 s. The test demonstrated acceptable reliability in previous studies and effectively discriminated between the different driver groups with varied driving experience [28,29].

### 2.3 Procedure

The experiment was administered by four postgraduates from four cities (Dalian, Shanghai, Zhengzhou, and Kunming) from July 1 to 15, 2019. After giving informed consent, participants completed a brief demographic questionnaire in which they were asked to provide information regarding age, sex, years of driving experience, total driving mileage, driving frequency after having obtained their driver’s license, and number of driving violations in the past year.

Prior to the experiment, the participants practiced with two video clips and answered the questions. During the formal experiment, participants were required to watch the 35 video clips displayed randomly on a 17-inch Lenovo computer screen with a resolution of 1024×720 pixels. Participants were given a maximum of 90 s to answer the corresponding questions after each clip. When the video clips stopped, the participants were asked to write down their answers on the questionnaire. The participants then completed the reaction time-based hazard perception test by pressing a mouse button when they detected a hazard, and the computer automatically recorded their reaction times. The entire experiment lasted approximately 50 minutes. Each participant received 60 RMB as compensation for participating in the study.

### 2.4 Data analyses

Data were analyzed in four steps using the statistical software SPSS 18.0. First, to avoid potential a ceiling or floor effect in the scores for each clip, descriptive statistics, such as the means, standard deviations, kurtosis and skewness of the total scores were analyzed. Items were deleted when kurtosis was greater than five or the mean approached the maximum score of six or the minimum score of zero [30]. As in previous studies [10,19,22], the total scores from all video clips were then submitted for separate classic item analysis and reliability analysis. Correlations between the scores for each clip and the total test scores were calculated, and video clips with a correlation coefficient of less than 0.3 were deleted. A minimum Cronbach’s alpha of 0.7 for the final test was expected in the reliability analysis. Second, the validity of the test was examined by analyzing the relationship between the total test scores and the reaction times as measured on the video-based hazard perception test. The missing data for reaction times for a specific video clip were replaced by the average score of all participants [3]. Third, a one-way ANOVA was conducted to compare the differences in the total test scores between novice and experienced drivers. A one-way ANCOVA was also used to examine the effect of other demographic factors on the total test scores. Finally, the differences in the test scores of drivers with traffic violations and those of drivers without traffic violations were examined to assess the external validity of the test.

## 3. Results

### 3.1 Video selection

Twelve items were deleted either because the kurtosis was greater than five (ten items) or the mean was approaching the minimum score (two items). Classic item analysis and reliability analysis indicated that an additional three items could be deleted to create a shorter test with improved reliability. Accordingly, the final test contained 20 video clips. The Cronbach’s alpha for the test was 0.862. The means, standard deviations and corrected item-total correlations are presented in Table 3.

**Table 3:** Mean, standard deviations and item-total correlations.

Clip no.	*Mean*	*SD*	Item test	Clip no.	*Mean*	*SD*	Item test
2	1.62	2.15	0.51	20	3.95	1.41	0.53
6	4.67	1.13	0.56	21	3.55	1.87	0.55
7	4.14	1.56	0.62	22	4.73	1.28	0.50
8	2.69	2.69	0.49	25	4.22	1.60	0.53
10	4.36	1.24	0.64	26	4.71	1.86	0.41
12	4.04	1.72	0.62	27	4.73	1.57	0.51
13	3.67	2.19	0.44	29	3.77	2.14	0.55
15	3.94	1.47	0.61	31	4.66	1.34	0.53
18	2.49	2.07	0.43	33	3.71	1.42	0.62
19	4.43	1.41	0.57	34	4.89	1.33	0.47

### 3.2 Validity of the test

The validity of the test was examined by analyzing the relationship between the total test scores and the reaction times as measured on the video-based hazard perception test. Significant correlations were found between the scores on the prediction test and the average reaction time (see Table 4). The findings provide evidence with respect to the discriminant validity of the test.

**Table 4:** Correlations between hazard prediction test scores, reaction time and traffic violations (*n* = 101).

Test score	Reaction time	Number of traffic violations
The average score for Q1	-0.20*	-0.29**
The average score for Q2	-0.22*	-0.13
The average score for Q3	-0.35**	-0.25*
Total test score	-0.32**	-0.28**

*p<0.05, two-tailed.

**p<0.01, two-tailed.

Q1 = question 1 “What is the hazard?”, Q2 = question 2 “Where is the hazard?”, Q3 = question 3 “What happens next?”, Reaction time = response time as measured on the video-based hazard perception test.

### 3.3 Test scores and demographic variables

A one-way ANOVA was conducted to examine the differences in total test scores between the two driver groups (see Table 5). Although the average total score was lower for novice drivers than it was for experienced drivers, *F*(1,99) = 81.92, *p*<0.01, $\eta_{p}^{2}$ = 0.453, it is possible that group differences on the test are due to other individual differences. When a one-way ANCOVA was performed with the total test score as the dependent variable, driving experience as the independent variable, and age, sex, and years of education as covariates, the results revealed that the effect of driving experience was still significant, *F*(1,96) = 9.75, *p*<0.01, $\eta_{p}^{2}$ = 0.092. Furthermore, while no significant differences based on sex, *F*(1,96) = 0.58, *p*>0.05, or years of education, *F*(1,96) = 0.02, *p*>0.05, were found, the effect of the covariate age was determined to be significant, *F*(1,96) = 12.50, *p*<0.01, $\eta_{p}^{2}$ = 0.115. The Pearson correlation analysis shows that age is positively correlated with the total test score (*r* = 0.67, *p*<0.01).

**Table 5:** Means and standard deviations for the hazard prediction test scores and reaction times (*M±SD*).

Variable	Novice (*n* = 54)	Experienced drivers (*n* = 47)	*F(1*, *99)*	*p*	$\eta_{p}^{2}$
The average score for Q1	1.24±0.32	1.62±0.36	33.08	0.001	0.250
The average score for Q2	1.32±0.19	1.63±0.25	48.47	0.001	0.329
The average score for Q3	0.88±0.29	1.40±0.39	57.90	0.001	0.369
Total test score	3.44±0.46	4.65±0.85	81.92	0.001	0.453
Reaction time	1.94±0.34	1.69 ±0.51	8.50	0.004	0.079

Q1 = question 1 “What is the hazard?”, Q2 = question 2 “Where is the hazard?”, Q3 = question 3 “What happens next?”, Reaction time = response time as measured on the video-based hazard perception test.

Novice drivers also exhibited slower reaction times to the hazards in the video clips than did experienced drivers, *F*(1, 99) = 8.50, *p*<0.01, $\eta_{p}^{2}$ = 0.079. However, the effect size was smaller than that of the average total score on the hazard prediction test.

### 3.4 Differences between drivers with or without traffic violations

Although the newly developed test exhibits acceptable reliability and validity, the present study further examined the association between the test scores and the number of traffic violations (see Table 4). As evidenced in Table 4, the results indicate that despite the score on question two, all other scores were significantly and negatively correlated with the number of traffic violations.

Next, a series of one-way ANCOVAs were conducted to further assess the discriminate validity of the test by comparing the differences in the test scores between drivers with traffic violations and drivers without traffic violations after controlling for demographic factors. Means and standard deviations for all measures are summarized in Table 6.

**Table 6:** Means and standard deviations for the test scores and reaction times (*M±SD*).

Variable	Drivers without violations (*n* = 78)	Drivers with violations (*n* = 23)	*F(1*, *94)*	*p*	$\eta_{p}^{2}$
The average score for Q1	1.45±0.36	1.29±0.45	4.03	0.048	0.041
The average score for Q2	1.49±0.25	1.39±0.31	0.84	0.362	0.009
The average score for Q3	1.17±0.41	0.97±0.45	3.28	0.073	0.034
Total test score	4.10±0.85	3.66±0.99	4.84	0.003	0.074

Q1 = question 1 “What is the hazard?”, Q2 = question 2 “Where is the hazard?”, Q3 = question 3 “What happens next?”.

The results indicate that drivers with traffic violations scored lower on question 1 and achieved lower total test scores than did drivers without traffic violations after controlling for demographic factors. In other words, because the newly developed test effectively distinguishes between driver groups, suggesting that its validity is acceptable.

## 4. Discussion

The aim of this study was to develop a hazard prediction test using the WHN method and examine its validity among the Chinese driving population. The findings suggest that the psychological property of the newly developed test is satisfactory and that it can be used for driver assessment and testing in China.

First, to develop a hazard prediction test using video clips filmed from drivers’ perspectives, the present study used the WHN method and asked drivers to answer questions after the screen was blacked out [10,19,22]. To ensure the ecological validity of the test, genuine video clips from natural driving environments were used, and all video clips were examined by expert drivers in terms of number of hazards, background complexity and video clarity, according to the criteria suggested in previous studies [3,5]. More importantly, the video clips contained as many hazard triggers as possible to better assess and reflect drivers’ hazard perception ability. After these scientific evaluations and selections were performed, the final test consisted of 20 video clips.

Second, the newly developed hazard prediction test exhibited acceptable reliability and validity. The reliability of the test was 0.862, suggesting that the test can be used in driver training courses, for driver assessments, and for driver testing. The present study determined experienced drivers outperformed novice drivers on the total score of the test. One explanation for this is that situation awareness develops as drivers gain more driving experience, and this experience allows drivers to know where to look in given situations and enables drivers to better anticipate other road users' driving behaviors. More importantly, the significant associations between the total scores of the test and the reaction times as measured on the video-based hazard perception test also indicated that the discriminant validity of the test was satisfactory. Notably, the test scores were more effective in distinguishing between drivers’ groups with varying driving experience than the video-based hazard perception test.

Furthermore, the present study examined the effects of other demographic variables on the test scores except driving experience. Although drivers’ experiences tend to increase as they grow older, experience-related differences in the total scores of the test were found after controlling for age and other demographic variables [3,5]. Therefore, it is of great importance to distinguish the effect of experience from that of age to better measure drivers’ hazard perception ability [5,31]. Notably, contradictory results have emerged in several studies, revealing no significant effects of age or experience on response latency [7]. One possible explanation for this contradiction is that some of these studies used abrupt hazards that triggered drivers' emergency response as experimental materials. Consistent with the findings of most previous studies [3,5], the present study found that the effects of neither sex nor education had a significant impact on the test scores.

Finally, this study found that drivers who had traffic violations had lower total scores on the test than did drivers who did not have traffic violations. Hence, the results suggest that the test is a highly useful tool for classifying driver groups. More importantly, the results indicate that drivers who had traffic violations may fail to correctly identify and predict hazards on the road, which, in turn, may pose an adverse impact on driving safety. Therefore, interventions or trainings targeting hazard perception ability may lead to positive outcomes [24]. Previous studies have shown that both young novice drivers and experienced drivers can benefit from systematic hazard perception training [32,33].

There are limitations in the present study. One limitation is that the participants were required to answer three sequential questions during the experiment. When the first question was incorrectly answered, the participant could correctly answer question two and/or question three. That is, the participants may realize that he/she did not answer question one correctly when he/she answered question two. Another limitation was that some participants may not correctly report their traffic violation history due to social interests. Thus, the associations between the test scores and traffic violations may be underestimated. However, the main purpose of the present study was to examine the reliability and validity of the newly developed hazard prediction test, and to that end, both factors were found to be satisfactory. To adjust for this weakness, future studies should use traffic violation records from police departments or insurance companies. Additionally, a follow-up study is needed to examine whether the test scores can predict drivers’ traffic violations or crashes over a six- or twelve-month period. The third limitation was that the number of participants was small. Although the present study tried to select participants from four representative cities in China, the sample may not represent the entire population of Chinese drivers. Hence, future studies should include a larger sample to investigate the external validity of the test.

To summarize, the present study developed a hazard prediction test based on the WHN method for Chinese drivers. The final test contained 20 video clips with satisfactory reliability. The significant associations between the test scores and reaction times as measured on the video-based hazard perception test indicated that the discriminant validity of the newly developed test was acceptable. More importantly, the test scores can effectively distinguish driver groups with different levels of driving experience and drivers with or without traffic violations. The hazard prediction test developed in the present study not only provides a reliable, valid and highly useful alternative for measuring Chinese drivers’ hazard perception ability but also makes it possible to incorporate this test into Chinese driver assessments and training.
